# GeneRIF indexing: sentence selection based on machine learning

**DOI:** 10.1186/1471-2105-14-171

**Published:** 2013-05-31

**Authors:** Antonio J Jimeno-Yepes, J Caitlin Sticco, James G Mork, Alan R Aronson

**Affiliations:** 1 National Library of Medicine, 8600 Rockville Pike, Bethesda, MD 20894, USA; 2 NICTA Victoria Research Lab, Melbourne VIC 3010, Australia

## Abstract

**Background:**

A Gene Reference Into Function (GeneRIF) describes novel functionality of genes. GeneRIFs are available from the National Center for Biotechnology Information (NCBI) Gene database. GeneRIF indexing is performed manually, and the intention of our work is to provide methods to support creating the GeneRIF entries. The creation of GeneRIF entries involves the identification of the genes mentioned in MEDLINE^®;^ citations and the sentences describing a novel function.

**Results:**

We have compared several learning algorithms and several features extracted or derived from MEDLINE sentences to determine if a sentence should be selected for GeneRIF indexing. Features are derived from the sentences or using mechanisms to augment the information provided by them: assigning a discourse label using a previously trained model, for example. We show that machine learning approaches with specific feature combinations achieve results close to one of the annotators. We have evaluated different feature sets and learning algorithms. In particular, Naïve Bayes achieves better performance with a selection of features similar to one used in related work, which considers the location of the sentence, the discourse of the sentence and the functional terminology in it.

**Conclusions:**

The current performance is at a level similar to human annotation and it shows that machine learning can be used to automate the task of sentence selection for GeneRIF annotation. The current experiments are limited to the human species. We would like to see how the methodology can be extended to other species, specifically the normalization of gene mentions in other species.

## Background

The large growth of the biomedical literature makes it difficult reading or simply identifying relevant information. For instance, MEDLINE ^*Ⓡ*^ growth is over 700k citations in 2011 [1]. There are efforts that investigate methods to support transferring the existing information in the biomedical literature to curated databases [2,3] or to index the biomedical literature [4-7]. Biocuration workflows are usually composed of the following main processing tasks [8-11]: (1) collecting related documents, (2) identifying and indexing entities of interest and, (3) collecting information for curating specific relations. A supporting tool for a given curation effort has to fulfill these tasks. In this work, we present research that we have performed to address the indexing of GeneRIF sentences.

A Gene Reference Into Function (GeneRIF) describes novel functionality of genes. The creation of GeneRIF entries involves the identification of the genes mentioned in MEDLINE citations and the citation sentences describing a novel function. GeneRIFs are available from the National Center for Biotechnology Information (NCBI) Gene database [12]. An example sentence is show below linked to the BRCA1 gene with gene id 672 from the citation with PubMed ^*Ⓡ*^ identifier (PMID) 22093627: FISH-positive EGFR expression is associated with gender status, but not correlated with the expression of ERCC1 and BRCA1 proteins in non-small cell lung cancer.

The Index Section [13] at the National Library of Medicine (NLM) has performed gene indexing since March 2002 [14]. NLM creates upwards of 80,000 GeneRIFs annually from articles indexed for MEDLINE.

GeneRIF indexing is performed manually, and the intention of our work is to provide a support tool similar to other indexing tools like the Medical Text Indexer (MTI) [4,15] already available at the NLM. The aim of the Gene Indexing Assistant (GIA) project at the NLM is to support creating the GeneRIF entries.

There is limited previous work related to GeneRIF span extraction. Most of the available publications are related to the TREC Genomics Track in 2003 [2]. There were two main tasks in this track, the first one consisted on identifying relevant citations to be considered for GeneRIF annotation. In the second task, the participants had to provide spans of text that would correspond to relevant GeneRIF annotations for a set of citations.

Considering this second task, the participants were not provided with a training data set. The Dice coefficient was used to measure the similarity between the submitted span of text from the title and abstract of the citation and the official GeneRIF text in the test set.

Surprisingly, one of the main conclusions was that a very competitive system could be obtained by just delivering the title of the citation as the best GeneRIF span of text. Few teams (EMC [16] and Berkley [17] being exceptions), achieved results better than that simple strategy. Another conclusion of the Genomics Track is that the sentence position in the citation is a good indicator for GeneRIF sentence identification, either the title or sentences close to the end of the citation.

Subsequent to the 2003 Genomics Track, there has been some further work related to GeneRIF sentence selection. Lu et al. [18,19] sought to reproduce the results already available from Entrez Gene (former name for the NCBI Gene database). In their approach, a set of features is identified from the sentences and used in the algorithm: Gene Ontology (GO) token matches, cue words and sentence position in the abstract. Gobeil et al. [20] combined argumentative features using discourse-analysis models (LASt) and an automatic text categorizer to estimate the density of Gene Ontology categories (GOEx). The combination of these two feature sets produces results comparable to the best 2003 Genomics Track system.

During a pre-analysis of this problem, we mapped the sentences already in the GeneRIF records in the NCBI Gene database to candidate sentences in the related MEDLINE abstracts, similar to [18]. The idea was to identify the relevant sentences in the abstract and to compare them to the other sentences that were not selected in order to identify rules for sentence extraction based on commonly occurring patterns. After the analysis of the mappings, one of our conclusions is that there are sentences in the abstract that could be suggested for GeneRIF indexing in addition to the sentences already indexed in the database. For instance, there is redundant information in the abstract and, in a small number of cases, genes not available in the NCBI Gene database will not have a GeneRIF sentence recorded for that abstract.

This is in contrast to previous work that focused on identifying the best span denoting a GeneRIF for a particular gene in a citation. These systems were evaluated comparing the overlap of the gold standard sentences based on the Dice coefficient. We find that this approach does not meet our needs since there is often more than one suitable sentence in the abstract. Our goal is to indicate, given a MEDLINE title and abstract, which sentences are more likely to be selected for GeneRIF annotation as well as additional alternative sentences. This motivated us to prepare a manually annotated gold standard for our work, including candidate sentences which were not selected in the original GeneRIF indexing.

Since there are cases where more than one GeneRIF sentence seems a plausible choice, the indexer will be presented with the top three options based on the confidence level returned by the system. The indexer is free to choose none, one, or more of the sentences to use in creating their GeneRIF. The current implementation of the GeneRIF annotation tool, includes a questionnaire screen with the tool recommended genes asking *Correct*, *Not a Gene*, *Wrong Species*, *Incorrect ID*, and *Irrelevant*. The information from the questionnaire is collected to improve the quality of the GeneRIF annotation tool and will likely be removed once full integration and implementation is completed.

The manual development of GeneRIF extraction rules did not prove to be very efficient in time and generalization of the prepared rules. We have explored the use of learning algorithms and several features extracted or derived from the sentences that might be combined to determine if it should be selected for GeneRIF indexing. In contrast to previous work, the features we use are derived from the sentences or using mechanisms to augment the information provided by them, e.g. assigning a discourse label using a previously trained model. The current study is limited to the human species. We would like to see how the methodology presented for GeneRIF sentence identification can be extended to other species. We have focused on humans for two reasons: 1) identifying genes in different species is non-trivial [21], and 2) the number of human GeneRIFs far surpasses the number of any other species. Humans make up over 61% of the GeneRIF entries, followed by *Mus Musculus* (Mouse) 18.82% and *Rattus norvegicus* (Rat) 6.95% (statistics based on the GeneRIFs available from NCBI ftp site [22]). By initially focusing on humans, we can provide more accurate recommendations for the largest segment of GeneRIF creation.

We show that machine learning approaches with a specific feature combination achieve performance close to annotators performance. The outcome of this work is being integrated into the current indexing support system.

The paper is organized as follows. In the next section, the development of our dataset is described and then the machine learning algorithms and features used in this work are presented. Then, we show the results, discuss their significance and suggest future work.

## Methods

In this section, we describe the methods, features and learning algorithms we used for GeneRIF sentence indexing. We describe as well the annotated data set that we have used in our experiments.

### GeneRIF data set

**Figure 1:**
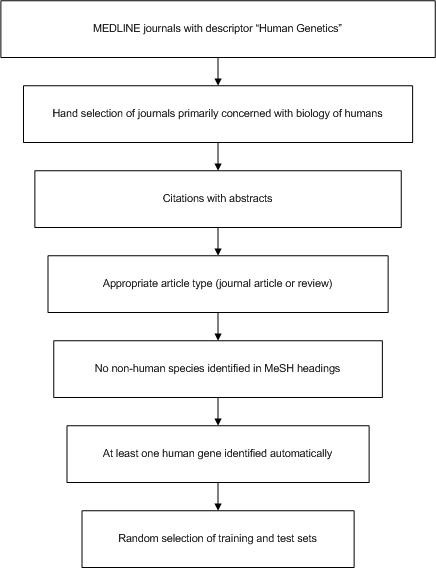
**Citation filtering pipeline.** The figure shows the citation filtering flow from all of MEDLINE to the training and testing sets.

We have developed a data set to compare and evaluate our GeneRIF indexing approaches. This is performed in two steps described below. As mentioned in the introduction, the current scope of our work is limited to the human species. The first step consists of selecting citations from human species journals. During the second step we apply Index Section rules for citation filtering plus additional rules to further focus the set of selected citations. Only articles from 2002 through 2011 from the 2011 MEDLINE Baseline [23] (11/19/2010) were used to build the data set. There was no GeneRIF indexing before 2002. Figure 1 shows the citation filtering pipeline.

#### Step 1: Journal selection for human genes

The journal selection is divided into a filtering step based on the journal subject and a manual selection, focusing on humans and genetics.

The steps to create the list of journals are: 

1. Selection of journals indexed for MEDLINE with the journal descriptor *Human genetics*. Journal Descriptors are broad categories used by the Bibliographic Services Division (BSD) to denote the research topics covered by a journal.

2. Manual removal of journals devoted to non-human species (e.g. Journal of experimental Zoology) and other journals primarily concerning gene therapy, genetic counseling, or ethics; e.g. “Law and the Human Genome Review".

3. From the set of journals found in the two previous steps, further journal filtering is performed based on the mention of common model species in their citations. Citations with mentions of 11 common model species terms are discarded: mouse, murine, yeast, fly, drosophila, cow, cattle, bovine, worm, c. elegans, and plant. Journals with fewer than 40% of their citations confounded by representative non-human species were selected to form our test bed.

#### Step 2: Citation filtering

Step 1 resulted in a total of 105,225 citations from 43 journals. Citations were selected from this set and then filtering as follows: 

1. Filter out citations that do not have an abstract.

2. Filter out citations denoting species other than humans based on the MeSH headings assigned to the citations.

3. Return citations with at least one gene mention. Gene mentions have been identified by a dictionary approach as described in the *Gene mention and normalization* section.

The rules presented below describe our implementation of the Index Section Rules [24]. The following filtering steps have been implemented based on the Index Section Rules: 

1. Keep review articles only if they focus on a particular gene. This means filtering out review citations with three or more different gene mentions.

2. Do not link news items, editorials or letters commenting on genes or proteins in another article. This is done by filtering by Publication Types *Journal Article*, *Meta-Analysis*, or *Review*.

3. Restrict to organisms in the taxonomic list for NCBI Gene database; we apply this rule to humans only.

After applying these rules, 23,518 citations were kept. In addition, the following rules require manual filtering of the citations. We are investigating ways of automating this processing for the production version of the system. 

1. Link a citation in which the basic biology of a gene from an in-scope organism is the primary point of the article. Do not create a link where the focus is genetic engineering, genetic databases, sample banks, population genetics unrelated to disease or function, and any topics other than the basic biology of the gene. In clinical articles, do not create links unless the focus of that article is some new aspect of that gene.

2. Do not create links for case reports with only a single patient.

Finally, applying this filtering, 373 citations were randomly selected for training and 151 citations for testing of the algorithms.

### Data set annotation

The above subset of filtered citations were collected for annotation.

The annotations were performed by two annotators. Guidelines were prepared and tested on a small set by the two annotators and refined before annotating the entire set.

The following items are available from the data set: 

1. Annotation of gene mentions. An initial annotation based on a dictionary approach is refined by the annotators. Identifiers from the NCBI Gene database are added to the gene mentions identified in the text.

2. Discourse annotations of the sentences. The following categories are used: Background, Conclusions, Methods, Purpose, Results, and Title.

3. Claim annotations of the sentences. The following categories are used: Established, Putative, and Non-Claim.

4. Annotation of GeneRIF categories of the sentences. Sentences have been labeled as either GeneRIF or non-GeneRIF sentences. From this annotation, the F-measure for the annotation agreement is 0.81. Since geneRIF sentences might have more distinctive characteristics, we performed a sub-categorization of the sentences, with the goal of assisting the learning algorithms. The GeneRIF sentences have been sub-categorized as: Expression, Function, Isolation, Other, Reference, and Structure. The F-measure of the agreement considering this set of categories is 0.60. Initial experiments with this sub-categorization showed that the learning algorithms had problems identifying these sentences, which might be due to the size of our corpus and the low agreement between the annotators on the sub-categorization.

Table 1 shows the distribution between GeneRIF and non-GeneRIF sentences in the training and testing sets.

**Table 1:** GeneRIF sentence distribution

**Set**	**Total**	**Positive**	**Negative**
Training	1987	829 (41.72%)	1158 (58.28%)
Testing	999	433 (43.34%)	566 (56.66%)

### Machine learning algorithms

For the experiments presented in this work, we have chosen a set of machine learning algorithms which are usually considered for text categorization problems. The algorithms used in our work are available from the Weka package [25] and are: decision tree (J48), Naïve Bayes (NB), Support Vector Machine (SVM) with linear kernel (we have used the Sequential minimal optimization (SMO) implementation) and AdaBoostM1 (using J48 as the base learner). Several feature sets evaluated in the experiments are presented in the Results and Discussion sections.

### Features

Previous work has proved the effectiveness of several features for GeneRIF indexing. For instance, Lu et al. [18] and Gobeil et al. [20] have shown that positional information, GO term annotation and argumentative features are relevant for GeneRIF indexing. In this section, we present the feature sets that we have explored during the training and testing of the learning algorithms.

#### Representation of the sentence text

Sentences are one source of features. Manual identification of indicative features has been used in Lu et al. [18]. The text of the citation sentences is turned into a bag-of-words prior to the training of a machine learning algorithm. Stop words have been removed based on Weka’s list, and tokens are lowercased. Given this pre-processing, we have performed tests with either unigrams or bigrams (determined by two consecutive tokens in the original text).

#### Sentence position information

**Figure 2:**
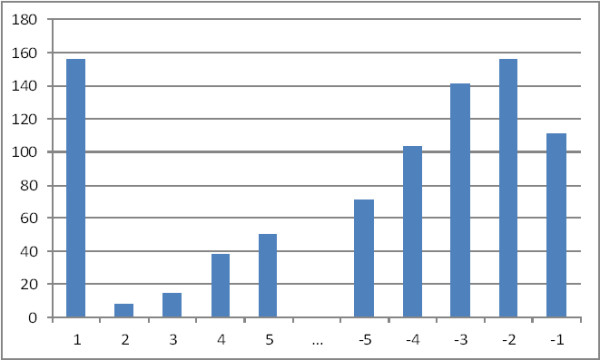
**GeneRIF and sentence distribution.** The y-axis denotes the number of GeneRIF sentences while the x-axis denotes the position of the sentence in the title and abstract of the citations. The position 1 is for the title, position 2 is the first sentence of the abstract and then increases by one. The negative numbers at the end of the x-axis denote the position of the sentence from the end of the abstract. The position -1 is for the last sentence of the abstract.

Sentence position in a given MEDLINE citation has shown to be very relevant for GeneRIF sentence identification. For example, in Genomics TREC 2003, a system delivering the title of the citation as GeneRIF sentence was already a competitive system. Lu et al. [18,19] extended the position information adding the position of the sentence starting from the end of the abstract. Considering our data set, we can see in Figure 2 the frequency of GeneRIFs in each position. The titles and the last sentences in the citation seem to contain a larger number of the GeneRIFs.

We have incorporated the position information into two features. The first one is the sentence number starting at the beginning of the citation.

The second feature is the total number of sentences minus the current sentence number, which allows us to identify the sentences closer to the end of the citation.

#### Sentence discourse feature

Gobeill et al. [20] have shown that argumentative features help in the identification of GeneRIF sentences. We have explored two candidate features as discourse annotation of the sentences. 

1. Our data set includes annotation about the discourse of the sentences. We have performed two experiments with this feature. First, we trained and tested the GeneRIF classifier with this feature as shown in the Results section. Since this annotation would not be available in a production system, we have trained a learning algorithm to identify these sentences. Results for this classifier show poor performance on this set, probably due to the small size of the training set, and thus it is not used.

2. Discourse labels for sentences are available from Structured Abstracts [26]. Structured Abstracts have assigned labels for the sections in the abstract of the MEDLINE citations. Currently, a quarter of the abstracts added to MEDLINE include section labels such as: BACKGROUND, OBJECTIVE, METHODS, RESULTS and CONCLUSIONS. We have collected one million citations with Structured Abstract labels. This set has been divided into 2/3 for training and 1/3 for testing. The features are the text of the sentence and the position of the sentence in the abstract. AdaBoostM1 from the MTI ML package [27] has been used due to the large size of the problem. One binary classifier per discourse label has been used.

3. Structured Abstracts seem to have a logical discourse structure that graphical models could use to improve discourse sentence annotation. This has been exploited in previous work [28,29], and we have trained a CRF model [30] based on Mallet [31] for Structured Abstract annotation. Due to the computational cost of this algorithm, we have limited the training and evaluation to 5,000 abstracts each.

#### Gene mention and normalization

Gene mention and normalization has been the focus of various challenges. Within the gene normalization task, dictionary approaches provide the link to existing resources. We have tried existing approaches like GNAT [32] on a set of abstracts relevant to our task in hand. The results in gene normalization performance were lower than we require. As part of the project, we have developed a gene mention and normalization approach that reuses available resources and algorithms.

We are interested in techniques that provide a mapping from gene mentions in text to NCBI Gene database entries. Thus we have prepared a dictionary for human gene names based on a filtered version of the NCBI Gene database and Online Mendelian Inheritance in Man (OMIM) that we have used with dictionary matching. We removed duplicates and filtered out certain misleading or ambiguous gene names, such as those ending with *disease*, *syndrome*, or *susceptibility*. The dictionary was then expanded with variants of each term to account for author preferences in punctuation and spacing. Variants were created for gene names that have a single dash in them. The variant generation algorithm creates a version replacing the dash with a space, and another variant with the dash simply removed. For example, *cortexin-2* would generate *cortexin 2* and *cortexin2* as variants. Variant generation will be improved in the next phase. One possibility will be to use one of the existing tools with a more advanced variant generation or identification algorithm.

Each resulting entry in the dictionary is linked to its originating NCBI Gene identifier. In the case of an entry that is common to multiple NCBI Gene records, all relevant identifiers are associated with the dictionary entry. The final list was then sorted into longest to shortest gene name order to facilitate identifying the longest possible matches in the text before identifying a component of the gene name. Sentences in an abstract are detected and tokenized using MetaMap [33].

Abbreviation resolution has been performed with MetaMap, which has reduced false positives significantly. MetaMap replaces locally defined abbreviations in the text with the original long form, using a Schwartz and Hearst [34] style algorithm to match long forms with parenthetical abbreviations. False positives are further reduced by using case-sensitive checks on mentions with common English homonyms and implementing domain-specific context rules. Although these modifications were successful, the extensive manual review required to make the latter two adjustments is not scalable to additional species or sustainable in genomes still being mapped.

Many gene names are ambiguous and may make reference to more than one gene identifier even within the human species. As of January 2012, 5,396 duplicate gene designations were included in 42,113 total Gene designations for humans. These designations include synonyms other than the official symbols and names used widely in the literature. However, these duplicates do include 52 official gene symbols. Just under 10% of our test set gene mentions are ambiguous and could refer to more than one Gene ID. These genes require disambiguation to normalize them to the correct identifier.

We tried two approaches to normalization. In the first one, we prioritized possible identifiers for a given mention according to a heuristic of *officialness*. For example, if one identifier matched the gene mention to an official name or symbol from the relevant organism naming authority, it was preferred over an identifier that matched a synonym or other alternate name.

In our second approach, we follow a method similar to Xu et al. [35]. In their work, for each gene a profile is given different types of information from MEDLINE abstracts. Information is extracted automatically and the abstracts are selected based on their annotation in the NCBI Gene database. The extracted information from the abstracts includes words, MeSH terms, UMLS concept identifiers, Gene Ontology terms and relations extracted using BioMedLEE [36].

We have implemented this approach using Gene entries to generate the profiles. The ambiguous cases are found based on an ambiguous term candidate list generated during the dictionary creation. When an ambiguous gene is found, the surrounding terms are used as context for their disambiguation. The context terms are used to generate a profile that is compared to the profile of the candidate Gene identifiers and the one closer to the context profile is selected.

#### Gene Ontology annotation

Gobeill et al. [20] have shown that the annotation of Gene Ontology (GO) contributes to the identification of GeneRIF sentences. We have used the EAGLi system [37] used by Gobeill et al. in their experiments and use the score as value for the GO annotation feature. This score is a numeric value that indicates the evidence of GO term in text based on [38].

## Results

In this section, we show the result of applying several features to several learning algorithms for the problem of finding GeneRIF sentences. Since we are interested in identifying the sentences instead of selecting the span of text with the message, as done in TREC Genomics 2003, the problem is similar to text categorization tasks. Sentences are labeled as relevant or not, and each combination of classifier and features is evaluated on how many sentences are identified correctly as relevant. Precision, recall and F-measure are used as evaluation measures. The overall results are shown in Table 2.

**Table 2:** GeneRIF prediction results

	**NB**			**SVM**			**ABM1**		
	**p**	**r**	**f**	**p**	**r**	**f**	**p**	**r**	**f**
pos	0.6052	0.3256	0.4234	0.6052	0.3256	0.4234	0.6594	0.7691	0.7100
posf	0.6705	0.5358	0.5956	0.6798	0.5196	0.5890	0.7218	0.7252	0.7235
text	0.5941	0.6051	0.5995	0.6322	0.6351	0.6336	**0.8250**	0.0762	0.1395
gene	0.5533	0.6952	0.6162	0.5533	0.6952	0.6162	0.5533	0.6952	0.6162
dis	0.6960	0.8037	0.7460	0.6755	0.8268	0.7435	0.7284	0.6628	0.6941
posf + dis	0.6974	0.8568	0.7689	0.6755	0.8268	0.7435	0.7323	0.7390	0.7356
posf + dis + gene	0.6996	0.8337	0.7608	0.6976	0.8152	0.7519	0.7875	0.7275	0.7563
posf + dis + go	0.6972	0.8614	**0.7707**	0.6755	0.8268	0.7435	0.7323	0.7390	0.7356
posf + dis + go + text	0.6751	0.7968	0.7309	0.7282	0.6559	0.6902	0.7342	0.7529	0.7434
disg	0.6061	**0.9630**	0.7440	0.6061	**0.9630**	0.7440	0.6061	**0.9630**	0.7440
posf + disg	0.6798	0.7552	0.7155	0.7250	0.7667	0.7452	0.7259	0.7644	0.7447
posf + disg + gene	0.7047	0.7991	0.7489	0.7886	0.7321	0.7593	0.7810	0.6836	0.7291
posf + disg + go	0.6708	0.7575	0.7115	0.7249	0.7667	0.7452	0.7259	0.7644	0.7447
posf + disg + go + text	0.6759	0.7321	0.7029	0.7802	0.6559	0.7127	0.7393	0.7206	0.7298

The results are show for each feature or their combination on the test set after training a Naïve Bayes (NB), Support Vector Machine (SVM) or AdaBoostM1 (ABM1). For each feature or feature combination, the precision (p), recall (r) and F-measure (f) are shown. The individual features are: sentence position from the beginning of the abstract (pos), sentence position from the end of the abstract (posf), text features from the sentence (text), gene mention and normalization (gene), discourse features predicted by the AdaBoostM1 classifier (dis), discourse features predicted by the CRF model (disg) and Gene Ontology score (go).

The position information (pos) alone shows good performance using AdaBoostM1 (0.71 F-measure). The performance is even slightly higher if we consider, in addition, the information of the position of the sentence relative to the end of the citation (posf) (0.72 F-measure).

The tokens extracted from the evaluated sentences (text) perform poorly compared to position information. In the case of AdaBoostM1, the F-measure decreases sharply while the precision is higher; this might be due to overfitting on the training set.

The gene mention feature (gene) achieves lower performance than that obtained with the position features (pos, posf). On the other hand, it performs better than using tokens from the text of the sentences (text).

As introduced in the Methods section, we have trained learning algorithms to enrich the sentences with discourse categories. We proposed two sources for the discourse, the annotation from our data set and annotation distilled from Structured Abstracts. We have trained classifiers on the position of the sentence and the tokens from the sentence. The discourse classifiers on the classes distilled from our data set achieve poor performance as we can see in Table 3, probably due to the reduced number of sentences available.

**Table 3:** GeneRIF data set discourse prediction

**Discourse**	**Positives**	**TP**	**FP**	**Precision**	**Recall**	**F-measure**
Background	258	180	78	0.7692	0.6977	0.7317
Conclusions	165	108	57	0.6316	0.6545	0.6429
Methods	179	105	74	0.5412	0.5866	0.5630
Purpose	36	24	12	0.3750	0.6667	0.4800
Results	260	163	97	0.6417	0.6269	0.6342

Results of a classifier trained on the discourse labels annotated in our data set. For each label we show the number of instances in the data set for each label (Positives), the number of True Positives (TP), the number of False Positives (FP) and the precision, recall and F-measure values.

On the other hand, the discourse classifier on Structured Abstracts shown in Table 4 has a better result. To this set, we have added the *Title* category to indicate that the sentence is the title of the paper. This discourse feature (dis) derived from the Structured Abstracts classifier is an even better indicator compared to the manual annotations. Despite of the CRF model (disg) performance on structured abstracts as shown in Table 5, the performance on GeneRIF indexing is lower compared to the AdaBoostM1 classifier.

**Table 4:** Structured abstracts discourse label prediction based on an AdaBoostM1 model

**Discourse**	**Positives**	**TP**	**FP**	**Precision**	**Recall**	**F-measure**
Background	18875	11045	8820	0.5560	0.5852	0.5702
Conclusions	53396	37402	12844	0.7444	0.7005	0.7218
Methods	85764	69003	21382	0.7634	0.8046	0.7835
Objective	26425	19237	7883	0.7093	0.7280	0.7185
Results	117546	93250	29424	0.7601	0.7933	0.7764

Results of an AdaBoostM1 classifier trained on structured abstracts. For each label we show the number of instances in the data set for each label (Positives), the number of True Positives (TP), the number of False Positives (FP) and the precision, recall and F-measure values.

**Table 5:** Structured abstracts discourse prediction label based on a CRF model

**Discourse**	**Positives**	**TP**	**FP**	**Precision**	**Recall**	**F-measure**
Background	6161	4154	2259	0.6477	0.6742	0.6607
Conclusions	10126	8455	1683	0.8340	0.8350	0.8345
Methods	15617	13473	2357	0.8511	0.8627	0.8569
Objective	4657	2810	1634	0.6323	0.6034	0.6175
Results	22228	18724	3240	0.8525	0.8424	0.8474

Results of a CRF trained model on structured abstracts. For each label we show the number of instances in the data set for each label (Positives), the number of True Positives (TP), the number of False Positives (FP) and the precision, recall and F-measure values.

If we combine the pos feature, which contains the position of the sentence from the beginning of the citation, the posf feature, which contains the position of the sentence from the end of the citation, and the dis feature, the performance improves considerably. Adding information from gene mentions annotation seems to make the performance drop. On the other hand, the performance with our gene ontology (GO) feature seems to improve the recall of the classifiers, which makes NB achieve the best performance on this set. Again, adding tokens from the sentences made the performance drop.

## Discussion

Results show that some of the selected features achieve good performance in sentence selection for GeneRIFs. The highest F-measure is obtained with NB and the following three features: posf, dis and go. The recall in this case is the highest as well: 0.86.

As found in previous work, the position of the sentence and the discourse of the sentence are already good indicators to categorize sentences according to their relevance to GeneRIF sentence selection. On the other hand, more specific features like the tokens from the sentence or even the features provided by gene mention do not achieve as good performance. In performing error analysis of our results, we have seen that the gene is not always explicitly mentioned in the relevant sentences. Instead, it is often referenced indirectly in other sentences.

Machine learning approaches did not produce results comparable to sentence position with the tokens extracted from the sentences. One possible explanation is that the tokens from the sentences are sparse and not generalizable for this task given the data set. On the other hand, the selected abstracts are related to genetic studies in humans which seem relevant to GeneRIF annotation. Then, the task is simply to identify where in the abstract the gene function is discussed or summarized. This might be in the title or at the end of the abstract, where the results and conclusions of the article are denoted.

Another reason for the low performance of the sentence tokens might be due to the small size of the data set. This is already an issue for the discourse classifier trained on our data set, compared to the one trained with Structured Abstracts annotation. Analyzing candidate tokens from the training sentences, we find that tokens like *significant*, *observed* and *novel* have high precision, between 0.75 and 0.8, but low recall, around 0.03. This is in contrast to previous work that used these features within their rule-based system [18]. The decision trees used in AdaBoostM1 would have identified relations among the features. On the other hand, identifying these relations seemed to achieve low performance. We believe that a larger set is needed to identify the relations between the tokens properly and avoid overfitting on a limited number of examples.

The GO annotation density alone achieves poor classification performance with all the classifiers. On the other hand, it helps improve the performance of NB with posf and dis features. We find this surprising since previous work has shown that GO annotation was relevant [20]. We find that the distribution scores are similarly distributed between GeneRIF (mean 15965.7 and standard deviation 25957.55) and non-GeneRIF sentences (mean 16553 and standard deviation 27401.1). Furthermore, we have a larger number of sentences which we have considered GeneRIF candidates, which could explain this difference.

Compared to the combinations of classifier and features, NB achieves the best performance using a selection of features: the position of the sentence from the beginning of the citation and from the end of it, the discourse label and the GO density. SVM achieves similar but lower performance compared to NB. SVM is high bias and low variance and might be more fitted to the data than NB or not as robust considering the differences between training and test data. In addition, the small number of independent features might give an advantage to NB compared to other classifiers. AdaBoostM1 achieves higher precision with lower recall. The results with SVM and AdaBoostM1 indicates that results might improve with a larger training set.

Our data set has labels for *claims* and *discourse* sentences. Even though we could not reproduce this annotation efficiently, we have evaluated a theoretical classifier performance using the annotation from the data set. The best result obtained with the *claims* sentence label and the best performing feature configuration is 0.8148 in the F-measure, obtained with AdaBoostM1. The best result obtained with the *discourse* sentence label and the best performing feature configuration is 0.8092 in the F-measure, obtained as well with AdaBoostM1. These results shows that improving the performance of the claims and discourse annotation algorithms would improve the performance of GeneRIF sentence selection.

Surprisingly, even though the performance in sentence classification in Structured Abstracts for the CRF model is much better compared to our previous model, we find that the classification of GeneRIF abstract sentences is not as good. One possible reason is that not all the GeneRIF abstracts have the same structure as compared to Structured Abstracts. Similar results have been identified in the literature [39]. This might explain why the model based on a set of discriminative binary classifiers could be performing better. Looking at the annotation performed by both classifiers, in some cases when an annotation was incorrect by the CRF model, there was no discourse annotation proposed by the discriminative one.

## Conclusions

In this work, we have evaluated different feature sets and learning algorithms for GeneRIF indexing. NB achieves its best performance with a selection of features similar to the features used in previous work [18,20]: the position of the sentence from the beginning of the citation and from the end of it, the discourse label and the GO density. We find as well that their combination is different compared to previous similar work. This might be explained by our focus on identifying candidate sentences from the title and abstract of the paper. Compared to the F-measure for inter-annotator agreement for GeneRIF indexing, our best results perform at a level similar to that of human annotation. This result is obtained by an optimal combination of the features based on learning algorithms. Current work is focused on humans, we would like to evaluate the performance of our approach to other species, which might require, among other things, adapting our gene mention and normalization algorithm.

We have found that performance is improved when adding information to the sentences (e.g. discourse and claim annotations).

Latent information derived from other types of annotation could be included and used with features extracted from the sentences. Topic discovery based on latent Dirichlet allocation [40] could be considered to augment the information in the sentences with topical information.

Even with the effort required to develop our data set, it is not enough to train a classifier based on sentence tokens or combinations of them. A larger data set might help in that regard. A larger manual effort would improve the result obtained by the learning algorithms. Annotation time could be reduced using active learning methods.

The data set used in the experiments is based on a selection of citations that is not fully automatic. Future work would involve providing a mechanism to automatically filter the citations following the definition in the Methods section in the citation filtering process. As we have seen during the development of the corpus, this requires several steps. We plan to further investigate the use of machine learning approaches to leverage the expansion of this work to cover a larger set of citations.

## Competing interests

The authors declare that they have no competing interests.

## Authors’ contributions

AJ designed and carried out the experiments, participated in the development of the methods and drafted the manuscript. JS designed the experiments, participated in the development of the data set and drafted the manuscript. JM designed the experiments, participated in the development of the methods and the data set and reviewed the manuscript. AA designed the experiments and reviewed the manuscript. All authors read and approved the final manuscript.
